# Dietary Selenium Influence on Milk Production, Blood Profiles, and Reproductive Efficiency in She-Camels and Neonatal Growth Performance

**DOI:** 10.1007/s12011-025-04639-5

**Published:** 2025-05-07

**Authors:** Mohamed S. Ayyat, Tarek H. Mostafa, Aya Saad Abd Elrasol ElTalawy, Mohammed E. R. Hammad, Ahmed A. Gabr, Adham A. Al-Sagheer

**Affiliations:** 1 Department of Animal Production, Faculty of Agriculture, Zagazig University, Zagazig, Egypt; 2 Camel Research Department, Animal Production Research Institute, Giza, Dokki Egypt; 3 Animal Production Department, Faculty of Agriculture, Tanta University, Tanta, Egypt

**Keywords:** Dromedary camel, Selenium, Body weight, Milk production, Protein and lipid metabolism

## Abstract

This study aimed to investigate the effects of dietary selenium supplementation during the pre- and post-partum periods on milk production, blood parameters, reproductive performance in she-camels, and the growth of their offspring. Twenty pregnant Maghrabi she-camels, in their last three months of gestation, were randomly divided into four experimental groups and supplemented with selenium-methionine at levels of 0, 0.2, 0.3, and 0.4 mg/kg diet. The experiment lasted 12 months, including a 3-month pre-partum and 9-month lactation period, during which the camels were fed a basal diet of concentrate feed, berseem hay, and rice straw. The results revealed that Se supplementation at all levels significantly increased placental weight and calf body weight at weaning, with the highest body weight and average daily gain observed in the 0.4 mg/kg group. Reproductive performance was also enhanced, with a reduction in postpartum first estrus interval and calving interval, particularly in the 0.4 mg/kg group. Milk production was significantly higher in Se-supplemented groups, with the 0.4 mg/kg group showing the longest lactation period and highest milk yield. Fat, lactose, and total solids yields improved, and Se concentration in milk increased with supplementation. Furthermore, Se supplementation led to improvements in blood biochemical parameters, including glucose, cholesterol, and triglycerides. Hormone concentrations of T3, T4, P4, and E2 were significantly higher in Se-supplemented groups. In conclusion, dietary Se supplementation during the pre- and post-partum periods can significantly enhance reproductive performance, milk production in she-camels and growth performance of their offspring, with the greatest benefits observed at 0.4 mg/kg.

## Introduction

Dromedary camels (*Camelus dromedarius*) are a vital livestock resource in arid and semi-arid regions, including the Middle East, Africa, and Asia [1, 2]. Renowned for their adaptability and endurance, they thrive in harsh environments characterized by extreme temperatures, scarce water resources, and limited vegetation, where other livestock struggle. According to the FAO, the global camel population is estimated at more than 42 million head, reflecting their critical role in providing food security and livelihoods for millions of people in these regions [3]. Camels are highly valued for their milk, a nutritious and easily digestible product that has been a dietary staple for centuries and is increasingly recognized for its health benefits and economic potential [4]. In recent years, the camel milk industry has gained significant attention, driving the transformation of camel farming from traditional nomadic practices to more modern systems, including intensive farming systems [5]. While these systems have contributed to higher milk production, they have also increased nutritional demands, necessitating optimized feeding strategies. Enhancing she-camel productivity through nutritional supplementation, especially with trace minerals, offers a promising approach to boost milk yield, improve metabolic functions, and support overall health [6].

Selenium (Se) is a vital trace element that plays a critical role in protecting cells from the harmful effects of hydroperoxides generated during cellular metabolism [7]. This protective function is carried out by selenoproteins, including the glutathione peroxidase (GSH-Px) family, thioredoxin reductases, and iodothyronine deiodinases. These proteins contain selenocysteine, an amino acid that is strategically positioned within the enzyme’s active site, enabling their antioxidant activity [8, 9]. Beyond its antioxidant properties, Se is also involved in modulating inflammation and supporting immune function [10].

In ruminants, supplementing Se at levels above nutritional requirements has been shown to mitigate oxidative stress caused by heat exposure in pregnant or lactating females [11]. It also reduces the expression of inflammatory genes [12] and influences colostrum volume and composition, including immunoglobulin G (IgG) levels [13]. Supplementing females with Se during pregnancy and lactation is an effective method for delivering Se to their offspring, as it passes through the placenta and is also present in colostrum and milk [13]. In cattle, Se deficiency has been linked to significant economic losses, including reduced fertility, retained placenta, and increased susceptibility to mastitis and metritis [14, 15]. The improvement in fertility associated with Se supplementation is thought to result from a reduction in embryonic mortality during early gestation. Additionally, Se supports immune function by aiding the development and activity of cytotoxic T cells, helper T cells, and natural killer cells [16, 17]. However, while Se supplementation provides significant benefits, excessive intake can be toxic, with doses exceeding 8 mg per day associated with selenosis, a condition characterized by clinical symptoms and histopathological lesions [18].

Recent studies suggest that supplementing diets with organic Se at 3.7 to 4.9 mg per head daily can increase milk yield and reduce somatic cell counts in milk [19]. While the National Research Council [20] recommends a dietary Se requirement of 0.3 mg/kg of dry matter for dairy cows, the specific requirements for camels have not been thoroughly established [21]. In practice, Se supplementation in camels is often based on cattle requirements, despite physiological differences between the species. Although extensive research has explored the effects of Se supplementation on lactation and reproductive performance in ruminants, studies focusing on camels are limited. Given the widespread use of Se in animal nutrition, further research into its metabolism and species-specific requirements in camels is essential.

Based on the established role of Se in enhancing antioxidant activity, immune function, and reproductive performance in ruminants, we hypothesized that dietary Se supplementation during the pre- and post-partum periods would improve milk production, blood parameters, reproductive performance in she-camels, and the growth performance of their offspring. This study aimed to investigate the effects of varying levels of dietary Se supplementation during the pre- and post-partum periods on these parameters in she-camels and their offspring.

## Materials and Methods

The research was carried out at the Center for Studies and Development of Camel Production, specifically at the Marsa Matrouh Station in Marsa Matrouh Governorate. This station operates under the Animal Production Research Institute (APRI), which is part of the Agricultural Research Centre located in Dokki, Giza, Egypt.

### Camels, Treatments, and Management

The study involved 20 Maghrabi she-camels (*C. dromedarius*), aged 6–9 years, weighing between 440 and 514 kg, and in their second or third parity. All camels were in the last three months of pregnancy, as confirmed via ultrasound, and had no history of peripartum diseases. Throughout the trial, all camels were monitored daily for health and welfare, with no mortality or significant health issues reported. The animals were randomly divided into four experimental groups of five camels each, based on body weight and parity. A 14-day acclimatization period was provided before the experiment began, during which the camels adapted to the experimental diet and housing conditions in semi-open pens. Following acclimatization, Se supplementation commenced three months before calving and continued for nine months postpartum. The control group (Group 1) received the basal diet without supplements, while Groups 2, 3, and 4 were provided with the basal diet supplemented with 0.2, 0.3, and 0.4 mg Se-methionine per kg of diet, respectively. Selenium supplementation was done by first mixing the Se-methionine (Sel-Plex®, Alltech Inc., Nicholasville, KY, USA) with 100 g of the diet, then thoroughly blending it into the remaining feed to ensure uniform distribution. The selenium concentrations used in this study were selected based on previous research evaluating Se supplementation in lactating ruminants [22, 23].

The camels were transferred to the maternity unit approximately 1–2 days before calving. All camels were fed a standardized basal diet formulated to meet their nutritional requirements during the three-month prepartum and nine-month postpartum periods [24, 25]. Before parturition, each camel received 8.5 kg of diet daily, and after calving, the diet was provided at 8 kg per animal. The composition of the basal diet for both periods is detailed in Table 1. Feeding occurred twice daily at 8 a.m. and 5 p.m., with unrestricted access to clean water. Samples of the unsupplemented diet were collected for chemical analysis, following the methods described by AOAC [26]. Calves were not directly supplemented with Se but received it through maternal transfer via colostrum and milk during the 7-month lactation period.

**Table 1:** Ingredients and chemical composition (g/kg feed) of the basal diet fed to she-camels during prepartum and postpartum periods

Item	Prepartum	Postpartum
**Ingredients**		
Berseem hay	235.2	312.5
Rice straw	235.3	250.0
Wheat bran	132.4	109.4
Yellow corn	132.4	109.4
Cotton seed meal	47.6	39.4
Barley	105.9	87.5
Rice bran	79.4	65.6
Molasses	15.9	13.1
Premix	10.6	8.8
Common salt	5.3	4.3
Total	1000	1000
**Chemical composition (g/kg DM)**^**1**^		
Organic matter	889.8	887.2
Crude fiber	171.2	191.2
Crude protein	105.7	101.2
Ether extract	32.4	28.9
Nitrogen-free extract^2^	580.5	565.9
Ash	110.2	112.8

^1^ Analyzed composition according to AOAC [26]

^2^ Calculated by difference as follows; Nitrogen-free extract = organic matter – (crude protein + ether extract + crude fiber)

### Milk Production and Milk Components

The camels were milked by hand twice daily, at 7 a.m. and 5 p.m. Milk yield was recorded by weighing the milk produced at each milking over two consecutive days each week throughout the nine-month of the lactation period and used to calculate the monthly milk yield. Composite milk samples, combining morning and evening milkings, were collected monthly from each animal for analysis of milk composition [6]. Daily milk samples were pooled proportionally based on yield, then divided into two subsamples: one preserved with bronopol (2-bromo- 2-nitropropane- 1,3-diol) for components analysis and the other for Se concentration [27]. Milk composition, including fat, protein, total solids, lactose, and ash, was analyzed using a MilkoScan (Model 133 B, Rajasthan Electronics & Instruments Limited, Rajasthan, India). The yields of total solids, solids-not-fat, protein, and lactose were calculated by multiplying total milk yield by their respective percentages [6].

### Analysis of Blood Samples and Selenium Concentration

Blood samples were collected from all camels in the study groups one month before and one month after parturition. The samples were collected in the morning prior to feeding. Blood was drawn from the jugular vein using heparinized vacuum tubes (Kuwaiti Egyptian for Medical Industries Co., El-Obour City, Egypt). Plasma was separated by centrifuging the blood at 3000 × *g* for 20 min and stored at − 20°C until further analysis. Plasma concentrations of total protein [28], glucose [29], cholesterol [30], triglycerides [31], and albumin [32] were measured, with globulin levels calculated by subtracting albumin from total protein. Hormone levels, including progesterone (P4), estradiol- 17β (E2), triiodothyronine (T3), and thyroxine (T4) were quantified using direct radioimmunoassay with Diagnostic Products Corporation kits (Los Angeles, USA), following the manufacturer’s instructions.

For Se analysis, blood and milk samples were digested with nitric acid (65%) and hydrogen peroxide (40%) and then heated until complete decomposition occurred [33]. Then, the samples were allowed to cool at room temperature. After cooling, the solution was filtered into a volumetric flask and diluted with deionized water for analysis. Selenium concentration was quantified using inductively coupled plasma mass spectrometry (ICP-OES, model 5100, Agilent, Santa Clara, CA).

### Evaluation of Reproductive Parameters in She-Camels

Reproductive performance during the postpartum period was assessed by monitoring several key indicators [6]. The first estrus was recorded when behavioral and physiological indicators, including general restlessness, aggressiveness, straddling of the hind legs, and swelling and discharge from the vulva, were first observed after parturition. The postpartum interval to first estrus (in days) was determined by daily observation of estrus-related behaviors. Days open were calculated as the duration from calving to successful conception, confirmed through estrus detection and pregnancy diagnosis. The number of services per conception was documented by counting the mating attempts needed until pregnancy was confirmed via ultrasound. The calving interval was defined as the time between two consecutive calvings. Placental expulsion was noted by observing the discharge of the placenta after birth, while uterine involution was evaluated using transrectal palpation and ultrasonography to monitor the reduction in uterine size until complete recovery.

### Body Weight Measurements and Colostrum Analysis

At the start of the experimental phase, the initial live body weight of the camels in all groups was recorded. Body weight was subsequently measured during the pre-partum period (three months prior to calving) and postpartum period (after calving). Additionally, the calves were weighed immediately after birth and every month until weaning at 7 months of age. Based on these measurements, the total and average daily weight gain of the calves was determined.

For the first week after birth, the calves were allowed to nurse from their mothers. After this period, the dams were transferred to the milking unit. Colostrum samples were collected from each dam within one hour of birth (first milking) and on the first, second, and third days postpartum for immunoglobulin analysis. The concentrations of immunoglobulins (IgG, IgM, and IgA) in the colostrum were quantified using bovine radial immunodiffusion (RID) kits, following the manufacturer’s instructions (Ltd, Birmingham, UK) and the methodologies outlined by Fahey and McKelvey [34] and Mancini et al. [35].

### Statistical Analysis

The data analyzed in this study included growth performance (body weight, average daily gain), reproductive parameters, milk production (yield, composition), blood biochemical profiles, hormonal concentrations (T3, T4, P4, E2), and immunoglobulin levels in colostrum (IgA, IgM, IgG). These data were subjected to ANOVA using the General Linear Model (GLM) procedures in the Statistical Analysis Software (SAS, 2004, version 8.0). Differences among the experimental groups were assessed at a significance level of 5%, and Duncan’s multiple range test was used to determine specific group differences. Results are reported as means ± SEM with corresponding p-values.

## Results

### Growth Performance of She-Camels and Newborn Calves

**Fig. 1:**
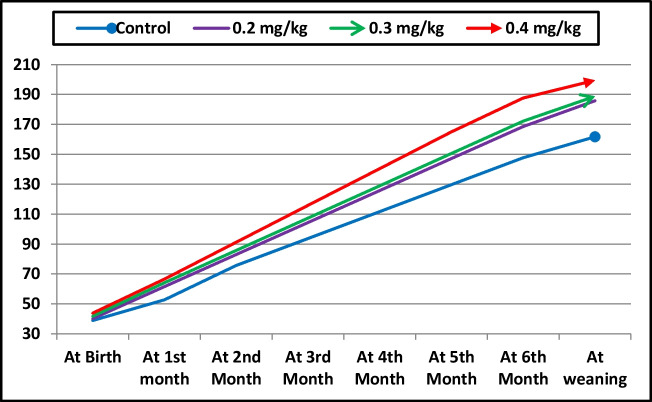
Weight of camel calves from birth to weaning as affected with dietary selenium supplementation in the diet of their mothers

Growth performance data of she-camels and their newborn calves are presented in Table 2. Selenium supplementation had no significant effect on she-camel weight before or after calving, fetal fluid weight, or calf birth weight. However, Se supplementation increased (P < 0.001) placental weight, with higher values observed in the 0.3 and 0.4 mg/kg groups compared with the control and 0.2 mg/kg groups. Calf growth parameters were positively influenced by Se supplementation. Throughout the trial period, calves supplemented with 0.4 mg/kg Se consistently achieved the highest body weight, followed by the 0.3 mg/kg and 0.2 mg/kg groups, while the control group had the lowest weight at weaning (Fig. 1). At weaning, calves from Se-supplemented groups had higher (P = 0.001) body weights compared with the control group. Similarly, average daily gain (ADG) improved (P = 0.001) in all Se-supplemented groups, with the highest ADG observed in the 0.4 mg/kg group (Table 2).

**Table 2:** Effect of selenium supplementation body weight of she-camels, weight of fetal fluids, weight of placenta and growth performance of newborn calves

Item	Control	Dietary selenium supplementation			P-value
		0.2 mg/kg	0.3 mg/kg	0.4 mg/kg	
She-camel weight before calving (kg)	481.80 ± 11.39	477.80 ± 5.054	477.20 ± 5.598	498.40 ± 5.085	0.177
She-camel weight after calving, kg	431.96 ± 8.36	425.42 ± 5.057	422.82 ± 4.948	441.48 ± 3.908	0.148
Weight of fetal fluids (kg)	9.34 ± 0.394	10.22 ± 0.448	10.22 ± 0.556	10.42 ± 0.596	0.457
Weight of placenta (kg)	1.62 ± 0.143^b^	2.00 ± 0.071^b^	2.42 ± 0.136^a^	2.68 ± 0.183^a^	< 0.001
**Average weight of born calves**					
At birth (kg)	38.88 ± 3.540	40.16 ± 3.082	41.74 ± 1.665	43.82 ± 1.567	0.585
At weaning (kg)	161.68 ± 5.195^b^	185.76 ± 4.531^a^	188.74 ± 3.322^a^	199.42 ± 7.103^a^	0.001
Average daily gain (kg)	0.77 ± 0.026^b^	0.88 ± 0.021^a^	0.90 ± 0.015^a^	0.95 ± 0.034^a^	0.001

Means in the same row with different superscripts are significantly different at P < 0.05

### Reproductive Performance of She-Camels:

As shown in Table 3, dietary Se supplementation reduced (P ≤ 0.001) the postpartum first estrus interval, with the shortest interval observed in the 0.4 mg/kg group, while the control group had the longest interval. The number of services per conception was significantly lower (P < 0.001) in Se-supplemented groups compared with the control. Days open decreased (P < 0.001) with Se supplementation, with the shortest interval observed in the 0.4 mg/kg group, followed by the 0.3 mg/kg and 0.2 mg/kg groups, while the control group exhibited the longest days open. Placental drop time was reduced (P = 0.004) in Se-supplemented groups compared with the control, with no significant differences among the 0.2, 0.3, and 0.4 mg/kg groups. Uterine involution time decreased (P < 0.001) with Se supplementation, with the fastest recovery observed in the 0.4 mg/kg group, while the control group showed the longest involution period. Calving interval was reduced (P < 0.001) with Se supplementation, with the 0.4 mg/kg group achieving the shortest interval, followed by the 0.3 mg/kg and 0.2 mg/kg groups, while the control group exhibited the longest interval.

**Table 3:** Effect of selenium supplementation on reproductive performance of she-camels

Item	Control	Dietary selenium supplementation			P-value
		0.2 mg/kg	0.3 mg/kg	0.4 mg/kg	
Postpartum 1 st estrus interval (d)	66.00 ± 2.665^a^	59.20 ± 4.091^ab^	54.00 ± 5.070^b^	39.40 ± 2.943^c^	0.001
Number of services per conception	4.40 ± 0.245^a^	3.00 ± 0.316^a^	2.60 ± 0.245^a^	2.40 ± 0.245^a^	< 0.001
Day open (d)	278.40 ± 1.887^a^	249.80 ± 4.727^b^	223.20 ± 6.507^c^	174.00 ± 4.147^d^	< 0.001
Placental drop (min)	175.00 ± 2.950^a^	161.20 ± 3.247^b^	153.60 ± 3.709^b^	151.80 ± 5.669^b^	0.004
Uterine involution (d)	47.60 ± 1.503^a^	42.40 ± 0.812^b^	39.60 ± 2.502^b^	28.40 ± 1.503^c^	< 0.001
Calving interval (d)	522.00 ± 3.886^a^	502.40 ± 4.844^b^	482.80 ± 4.116^c^	448.20 ± 6.461^d^	< 0.001

Means in the same row with different superscripts are significantly different at P < 0.05

### Milk Production and Composition

**Fig. 2:**
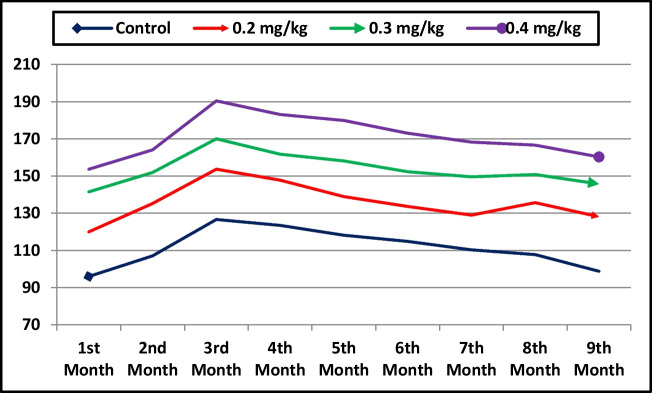
Monthly milk yield (kg) of she-camels supplemented with selenium in their diet (0.2, 0.3 and 0.4 mg/kg) and in the control group

The effects of dietary treatment on the milk yield and composition are given in Table 4. Dietary Se supplementation significantly increased (P < 0.001) the lactation period, total milk yield, and daily milk yield, with the longest lactation period and highest milk yield observed in the 0.4 mg/kg group, followed by the 0.3 mg/kg and 0.2 mg/kg groups, while the control group exhibited the shortest lactation period and lowest milk yield. Compared with the control group, Se supplementation led to a significant increase in monthly milk yield, with the highest values observed in the 0.4 mg/kg group throughout lactation (Fig. 2). Similarly, fat yield, lactose yield, total solids yield, and solids-not-fat yield increased (P ≤ 0.008) with Se supplementation, with the highest values recorded in the 0.4 mg/kg group. Protein yield showed a trend toward being higher in the Se-supplemented groups compared to the control (P = 0.085). Milk composition parameters, including fat, protein, lactose, total solids, and solids-not-fat percentages, were not significantly affected by Se supplementation. However, ash content increased (P = 0.018) with Se supplementation, with the highest value recorded in the 0.4 mg/kg group. Selenium concentration in milk increased (P = 0.013) with dietary Se supplementation, with the highest concentration observed in the 0.4 mg/kg group, followed by the 0.3 mg/kg and 0.2 mg/kg groups, while the control group had the lowest concentration.

**Table 4:** Effect of selenium supplementation on milk yield and milk composition of She-camels

Item	Control	Dietary selenium supplementation			P-value
		0.2 mg/kg	0.3 mg/kg	0.4 mg/kg	
**Milk yield**					
Lactation period (d)	265.60 ± 8.88^c^	308.00 ± 7.01^b^	333.20 ± 9.51^ab^	347.80 ± 14.12^a^	< 0.001
Total milk yield (kg)	1002.7 ± 19.28^c^	1221.8 ± 47.50^b^	1381.7 ± 69.11^ab^	1539.4 ± 72.27^c^	< 0.001
Daily milk yield (kg)	3.71 ± 0.07^c^	4.53 ± 0.18^b^	5.12 ± 0.26^ab^	5.58 ± 0.29^a^	< 0.001
Fat yield (kg)	43.62 ± 3.44^c^	56.93 ± 5.50^bc^	68.25 ± 6.95^ab^	79.10 ± 8.50^a^	0.008
Protein yield (kg)	40.43 ± 5.38	52.00 ± 9.31	61.20 ± 6.54	71.37 ± 10.26	0.085
Lactose yield (kg)	45.55 ± 3.14^c^	58.31 ± 3.92^bc^	70.70 ± 7.61^ab^	80.04 ± 6.31^a^	0.003
Total solids yield (kg)	137.96 ± 10.11^c^	177.67 ± 6.57^bc^	212.64 ± 16.24^ab^	244.96 ± 20.77^a^	0.001
Solids not fat yield (kg)	94.34 ± 8.25^c^	120.74 ± 7.14^bc^	144.39 ± 9.84^ab^	165.86 ± 13.69^a^	< 0.001
**Milk Composition**					
Fat %	4.34 ± 0.30	4.64 ± 0.32	4.90 ± 0.23	5.14 ± 0.48	0.421
Protein %	4.04 ± 0.53	4.27 ± 0.72	4.45 ± 0.48	4.62 ± 0.58	0.910
Lactose %	4.54 ± 0.28	4.83 ± 0.45	5.07 ± 0.30	5.19 ± 0.28	0.539
Ash %	0.84 ± 0.03^c^	0.85 ± 0.02^bc^	0.91 ± 0.02^ab^	0.94 ± 0.01^a^	0.018
Total solids %	13.75 ± 0.93	14.59 ± 0.58	15.32 ± 0.45	15.88 ± 0.99	0.270
Solids not-fat %	9.41 ± 0.80	9.95 ± 0.74	10.43 ± 0.34	10.74 ± 0.60	0.505
Selenium (ppm)	0.42 ± 0.07^c^	0.48 ± 0.05^bc^	0.76 ± 0.06^ab^	0.88 ± 0.17^a^	0.013

Means in the same row with different superscripts are significantly different at P < 0.05

### Immunoglobulins in Colostrum

Colostrum immunoglobulin concentrations of she-camels during the first three postpartum days are shown in Table 5. Dietary Se supplementation significantly increased (P < 0.001) colostrum IgA, IgM, and IgG concentrations across all three days. For IgA, supplementation with 0.4 mg/kg Se resulted in the highest concentrations on all three days (P < 0.001), while the 0.3 mg/kg and 0.2 mg/kg groups had intermediate values, and the control group had the lowest levels. Similarly, IgM concentrations increased (P ≤ 0.001) with Se supplementation, with the highest levels recorded in the 0.4 mg/kg group, followed by the 0.3 mg/kg and 0.2 mg/kg groups, while the control group had the lowest values. Colostrum IgG concentrations were significantly higher (P < 0.001) in Se-supplemented groups compared with the control group on all three days. The highest values were observed in the 0.4 mg/kg group, followed by the 0.3 mg/kg and 0.2 mg/kg groups, while the control group had the lowest concentrations.

**Table 5:** Effect of selenium supplementation on colostrum immunoglobulins concentrations of she-camels on the first three postpartum days

Item	Control	Dietary selenium supplementation			P-value
		0.2 mg/kg	0.3 mg/kg	0.4 mg/kg	
**IgA (mg/ml)**					
Day 1	3.68 ± 0.24^c^	7.01 ± 0.63^b^	8.77 ± 0.67^b^	11.75 ± 1.02^a^	< 0.001
Day 2	3.79 ± 0.24^c^	6.17 ± 0.45^b^	8.13 ± 0.76^b^	11.15 ± 1.10^a^	< 0.001
Day 3	3.38 ± 0.52^c^	5.13 ± 0.56^c^	7.12 ± 0.49^b^	9.140 ± 0.96^a^	< 0.001
**IgM (mg/ml)**					
Day 1	5.00 ± 0.29^c^	7.36 ± 0.61^b^	9.93 ± 0.87^a^	11.10 ± 0.90^a^	< 0.001
Day 2	4.60 ± 0.42^c^	6.35 ± 0.59^bc^	8.38 ± 0.73^ab^	9.79 ± 1.16^a^	0.001
Day 3	3.87 ± 0.44^b^	5.14 ± 0.45^b^	7.10 ± 0.55^a^	8.16 ± 0.74^a^	< 0.001
**IgG (mg/ml)**					
Day 1	34.60 ± 0.94^d^	46.10 ± 0.97^c^	50.60 ± 1.12^b^	55.10 ± 1.93^a^	< 0.001
Day 2	33.00 ± 0.85^c^	43.40 ± 1.02^b^	48.10 ± 0.95^ab^	51.40 ± 3.59^a^	< 0.001
Day 3	29.58 ± 0.43^c^	41.40 ± 1.11^b^	45.40 ± 1.48^b^	49.80 ± 1.90^a^	< 0.001

Means in the same row with different superscripts are significantly different at P < 0.05

### Blood Biochemical Parameters

The effect of Se supplementation on pre- and post-partum blood plasma biochemical concentrations in lactating she-camels is presented in Table 6. One month pre-partum, total protein concentration increased (P = 0.013) with Se supplementation, with the highest value observed in the 0.4 mg/kg group. Glucose concentration also increased (P = 0.012) with Se supplementation, with significantly higher values in all supplemented groups compared with the control. Similarly, cholesterol (P = 0.003) and triglyceride (P < 0.001) concentrations were elevated in Se-supplemented groups. Plasma Se concentrations showed a significant increase (P < 0.001) with increasing dietary Se levels. However, no significant differences were observed in albumin (P = 0.152) and globulin (P = 0.823) concentrations among the groups.

**Table 6:** Effect of selenium supplementation on pre- and post-partum concentrations of some biochemical parameters in blood plasma of lactating she-camels

Item	Control	Dietary selenium supplementation			P-value
		0.2 mg/kg	0.3 mg/kg	0.4 mg/kg	
**One month pre-partum:**					
Total proteins (g/dl)	6.94 ± 0.39^c^	7.21 ± 0.32^bc^	7.97 ± 0.20^ab^	8.49 ± 0.32	0.013
Albumin (g/dl)	3.00 ± 0.33	3.19 ± 0.41	3.83 ± 0.33	4.06 ± 0.35	0.152
Globulin (g/dl)	3.94 ± 0.26	4.02 ± 0.35	4.13 ± 0.50	4.43 ± 0.41	0.823
Glucose (mg/dl)	52.86 ± 1.36^b^	62.42 ± 3.20^a^	65.60 ± 2.82^a^	68.48 ± 4.07	0.012
Cholesterol (mg/dl)	78.92 ± 2.54^b^	94.14 ± 4.03^a^	95.62 ± 1.73^a^	97.66 ± 4.03	0.003
Triglycerides (mg/dl)	105.40 ± 1.21^c^	110.00 ± 2.17^bc^	116.20 ± 2.31^ab^	123.00 ± 3.89	< 0.001
Selenium (µg/l)	2.38 ± 0.39^c^	4.48 ± 0.36^b^	6.27 ± 0.56^a^	7.52 ± 0.71	< 0.001
**One month postpartum:**					
Total proteins (g/dl)	7.21 ± 0.50	7.67 ± 0.21	8.12 ± 0.17	8.57 ± 0.44	0.082
Albumin (g/dl)	3.25 ± 0.47	3.59 ± 0.24	3.90 ± 0.41	4.20 ± 0.50	0.440
Globulin (g/dl)	3.96 ± 0.58	4.08 ± 0.26	4.22 ± 0.48	4.57 ± 0.63	0.844
Glucose (mg/dl)	51.82 ± 1.93^c^	63.96 ± 3.13^b^	68.92 ± 2.74^ab^	73.42 ± 3.85^a^	0.001
Cholesterol (mg/dl)	79.80 ± 2.31^b^	95.94 ± 4.19^a^	98.98 ± 1.84^a^	102.56 ± 4.14^a^	0.001
Triglycerides (mg/dl)	106.00 ± 1.38^b^	119.12 ± 2.17^a^	120.38 ± 4.61^a^	126.10 ± 2.62^a^	0.002
Selenium (µg/l)	3.16 ± 0.35^d^	6.32 ± 0.58^c^	9.14 ± 0.70	12.13 ± 0.74^a^	< 0.001

Means in the same row with different superscripts are significantly different at P < 0.05

One month postpartum, Se supplementation led to increased glucose (P = 0.001), cholesterol (P = 0.001), and triglyceride (P = 0.002) concentrations, with the highest values recorded in the 0.4 mg/kg Se group. Plasma Se concentrations also increased (P < 0.001) with higher Se inclusion levels. However, no significant differences were found in total protein (P = 0.082), albumin (P = 0.440), and globulin (P = 0.844) concentrations among the groups.

### Hormonal Concentration

As presented in Table 7, T3 concentration increased significantly (P < 0.001) with Se supplementation, with the highest values observed in the 0.4 mg/kg group. A similar trend was noted postpartum (P < 0.001), where Se supplementation elevated T3 levels compared with the control. Thyroxine concentration showed no significant differences prepartum (P = 0.078), whereas postpartum, Se supplementation significantly increased T4 levels (P < 0.001), reaching the highest concentration in the 0.4 mg/kg group. Also, P4 concentration was significantly elevated prepartum (P < 0.001) and postpartum (P = 0.004) with Se supplementation. The highest P4 levels were recorded in the 0.4 mg/kg group both prepartum and postpartum. Similarly, E2 concentration increased significantly prepartum (P < 0.001) with Se supplementation, with a marked rise in the 0.4 mg/kg group. Postpartum, E2 concentration also increased (P = 0.002), with the highest values observed in Se-supplemented groups.

**Table 7:** Effect of selenium supplementation on plasma concentration of thyroid and ovarian hormones of she-camels

Item	Control	Dietary selenium supplementation			P-value
		0.2 mg/kg	0.3 mg/kg	0.4 mg/kg	
**T**_**3**_** (ng/dl)**					
Prepartum	99.16 ± 4.61^c^	114.50 ± 3.15^b^	123.58 ± 3.165^ab^	129.64 ± 3.34^a^	< 0.001
Postpartum	100.34 ± 4.77^c^	116.96 ± 3.27^b^	127.50 ± 3.202^ab^	134.90 ± 3.31^a^	< 0.001
**T**_**4**_** (ng/dl)**					
Prepartum	3.80 ± 0.54	5.64 ± 0.81	6.94 ± 1.589	8.84 ± 1.76	0.078
Postpartum	4.92 ± 0.63^d^	7.88 ± 0.58^c^	12.74 ± 0.840^b^	18.00 ± 0.82^a^	< 0.001
**P**_**4**_** (ng/dl)**					
Prepartum	4.32 ± 0.55^d^	6.98 ± 0.70^c^	8.62 ± 0.450^b^	10.44 ± 0.35^a^	< 0.001
Postpartum	3.09 ± 0.55^c^	4.38 ± 0.50^bc^	5.22 ± 0.376^ab^	6.60 ± 0.77^a^	0.004
**E**_**2**_** (pg/dl)**					
Prepartum	43.92 ± 1.35^d^	68.34 ± 6.00^c^	97.04 ± 4.70^b^	115.18 ± 3.33^a^	< 0.001
Postpartum	42.54 ± 3.87^c^	53.92 ± 6.20^bc^	68.40 ± 7.09^ab^	78.24 ± 4.41^a^	0.002

Means in the same row with different superscripts are significantly different at P < 0.05

## Discussion

This study found no significant difference in the body weight of she-camels across varying Se levels during the pre- or post-partum periods. However, it demonstrated that Se supplementation in she-camels during pre- and post-parturition significantly improved calf growth, as evidenced by increased body weight at weaning and ADG. This positive effects can be attributed to increased Se levels in milk, enhanced immunoglobulin concentrations in colostrum observed in this study, which contributed to improved passive immunity transfer and greater disease resistance. Similar findings have been reported in newborn calves up to 70 days or five months of age when cows were supplemented with Se [36, 37] However, several studies indicate that organic Se supplementation does not influence calf growth performance [38, 39]. Mehdi and Dufrasne [17] suggested that while Se does not directly stimulate growth in calves, it aids in eliminating factors that could hinder or delay their growth. Blood Se concentration in she-camels was higher in supplemented groups and declined before parturition, particularly in the control group. This decline suggests an increased demand for Se during late gestation due to fetal requirements and colostrum formation [40]. Selenium is a crucial trace element for fetal development, particularly during the last trimester of pregnancy. Its role encompasses antioxidant protection, regulation of the cell cycle, and support for tissue development. Maternal Se levels significantly influence fetal health, with increased requirements noted as gestation progresses [41, 42]. This is primarily due to the fetus’s accumulation of selenium, which is essential for various biological functions [43]. Additionally, cows fed Se-supplemented diets during the dry period showed increased serum Se concentrations in their calves at birth, supporting the role of maternal nutrition in neonatal Se status [44]. Additionally, Se’s antioxidant properties help maintain colostrum’s redox balance, further optimizing immunity transfer [45].

This study demonstrated that Se supplementation positively influenced the reproductive performance of she-camels, leading to significant reductions in postpartum estrus interval, number of services per conception, days open, placental drop time, uterine involution time, and calving interval. Selenium plays a crucial role in the function of GSH-Px, an enzyme essential for combating oxidative stress, which is particularly harmful during the peripartum period. In camels, Se supplementation has been shown to enhance GSH-Px activity, helping to protect reproductive tissues from oxidative damage and supporting postpartum recovery [46]. Previous studies have reported a direct correlation between Se supplementation and increased serum Se concentrations, as well as elevated GSH-Px activity, which contribute to improved metabolic stability during both lactation and gestation [46, 47]. This antioxidant effect likely explains the observed reductions in uterine involution time and placental drop time in this study, as oxidative stress is known to delay tissue repair and increase the risk of retained placenta [48, 49].

The observed reduction in postpartum estrus intervals and services per conception is consistent with findings in cattle, where Se supplementation, often in combination with vitamin E, has been shown to improve ovarian cyclicity, enhance progesterone production, and accelerate the resumption of estrous cycles after parturition [49, 50]. Additionally, Se plays a significant role in thyroid hormone metabolism, which influences energy balance and reproductive cyclicity [48]. Moreover, the reduction in days open and calving intervals observed in this study is supported by previous research demonstrating that Se supplementation enhanced overall metabolic health, indirectly supporting faster return to estrus and successful conception. For instance, camels supplemented with organic Se exhibited higher colostrum quality and neonatal survival rates, factors that reduce maternal metabolic strain and facilitate quicker reproductive recovery [46, 51]. Similarly, in cattle, Se-deficient herds showed prolonged calving intervals due to subclinical metabolic disorders, which were mitigated by targeted supplementation [48, 49].

Selenium supplementation in she-camels significantly improves milk yield and certain components of milk composition, particularly the yields of fat, lactose, total solids, and solids not fat. However, the percentages of fat, protein, lactose, and total solids in milk did not show significant changes with Se supplementation. In camels, Se supplementation has been shown to elevate serum and milk Se concentrations, improving metabolic resilience and nutrient partitioning toward milk synthesis [21, 46]. The increased milk yield in this study may reflect enhanced energy metabolism and reduced oxidative damage to mammary epithelial cells, enabling greater secretory activity without compromising component synthesis rates [46, 48]. Similar results were reported in dairy cows, where organic Se supplementation increased milk production and Se bioavailability while maintaining milk composition ratios [52, 53]. The stability of fat, protein, and lactose percentages in camel milk despite higher yields parallels findings in other ruminants. For example, studies on cattle demonstrated that Se supplementation did not alter fat, lactose or protein proportions, as these are tightly regulated by genetic and homeostatic mechanisms [52].

The significant increase in IgA, IgM, and IgG concentrations in colostrum following Se supplementation is consistent with studies on other ruminants. For instance, cows receiving Se supplements had significantly higher IgG concentrations in colostrum compared to non-supplemented cows [54]. Hall et al. [55] reported that providing supranutritional Se supplementation to dairy cows enhances colostral Se levels, thereby improving IgG passive transfer and overall health in newborn calves. The mechanism likely involves Se ‘s role in enhancing the activity of selenoproteins, which are critical for immune function and antioxidant defense [56]. Another possible explanation is that supranutritional Se supplementation could affect the number of specific nutrient transporters or the growth and blood vessel formation in mammary tissues, similar to what is suggested for intestinal tissues [57].

The observed increases in total proteins, glucose, cholesterol, and triglycerides in the blood plasma of Se-supplemented she-camels can be attributed to Se’s role in metabolic regulation. Selenium serves as the active center in selenoproteins and plays a key role in glucose metabolism and redox processes in animals [58]. The elevation in cholesterol and triglycerides may also reflect Se’s influence on lipid metabolism, as seen in studies where Se supplementation improved lipid profiles in adults, albeit with some variability in outcomes [59, 60]. The significant increase in blood concentrations of T3, T4, P4, and E2 in Se-supplemented groups underscores Se’s role in endocrine regulation [61]. Selenium is essential for the synthesis and metabolism of thyroid hormones (T3 and T4), as it is a component of iodothyronine deiodinases, which convert T4 to the more active T3 [62]. Studies have shown that Se supplementation significantly elevates serum T3 levels in sheep, particularly when provided in organic forms [63]. Higher Se levels are also associated with increased activity of glutathione peroxidase, an enzyme that protects the thyroid gland and supports hormone synthesis [64]. Beyond its impact on thyroid hormones, Se influences reproductive hormones such as P4 and E2 in ruminants, likely by enhancing overall metabolic health and reproductive efficiency. Additionally, research suggests that Se enhances reproductive hormone secretion, potentially by improving blood flow and nutrient delivery to reproductive organs [65].

## Conclusion

Dietary organic Se supplementation during the pre- and post-partum periods had a positive effect on offspring growth performance, she-camel reproductive performance, and milk production parameters. A selenium supplement level of 0.4 mg/kg produced the most significant improvements in these areas, including increased milk yield, improved average daily calf gain, and improved reproductive efficiency. In addition, Se supplementation increased colostrum immunoglobulin levels and blood biochemical parameters, demonstrating its beneficial role in supporting maternal and offspring health. Further studies are needed to investigate the underlying metabolic mechanisms and optimize Se supplementation strategies in camels.

## Data Availability

The data that support the findings of this study are available from the corresponding author upon reasonable request.
